# Overexpression of TNFSF11 reduces GPX4 levels and increases sensitivity to ferroptosis inducers in lung adenocarcinoma

**DOI:** 10.1186/s12967-024-05112-y

**Published:** 2024-04-09

**Authors:** Zizhen Li, Wenhua Lu, Feng Yin, Peiting Zeng, Heping Li, Amin Huang

**Affiliations:** 1 Department of Medical Oncology, The First Affiliated Hospital, Sun Yat-Sen University, Guangzhou, 510000 China; 2 State Key Laboratory of Oncology in Southern China, Sun Yat-Sen University Cancer Center, Guangzhou, 510000 China; 3 Department of Hematology, Sun Yat-sen Memorial Hospital, Sun Yat-sen University, Guangzhou, 510120 China

**Keywords:** LUAD, TNFSF11, GPX4, Ferroptosis, Erastin, RSL3

## Abstract

**Background:**

Lung adenocarcinoma (LUAD), the most common and lethal subtype of lung cancer, continues to be a major health concern worldwide. Despite advances in targeted and immune therapies, only a minority of patients derive substantial benefits. As a result, the urgent need for novel therapeutic strategies to improve lung cancer treatment outcomes remains undiminished.

**Methods:**

In our study, we employed the TIMER database to scrutinize TNFSF11 expression across various cancer types. We further examined the differential expression of TNFSF11 in normal and tumor tissues utilizing the TCGA-LUAD dataset and tissue microarray, and probed the associations between TNFSF11 expression and clinicopathological parameters within the TCGA-LUAD dataset. We used the GSE31210 dataset for external validation. To identify genes strongly linked to TNFSF11, we engaged LinkedOmics and conducted a KEGG pathway enrichment analysis using the WEB-based Gene SeT AnaLysis Toolkit. Moreover, we investigated the function of TNFSF11 through gene knockdown or overexpression approaches and explore its function in tumor cells. The therapeutic impact of ferroptosis inducers in tumors overexpressing TNFSF11 were also investigated through in vivo and in vitro experiments. Through these extensive analyses, we shed light on the potential role of TNFSF11 in lung adenocarcinoma, underscoring potential therapeutic targets for this malignancy.

**Results:**

This research uncovers the overexpression of TNFSF11 in LUAD patients and its inverse correlation with peroxisome-related enzymes. By utilizing gene knockdown or overexpression assays, we found that TNFSF11 was negatively associated with GPX4. Furthermore, cells with TNFSF11 overexpression were relatively more sensitive to the ferroptosis inducers.

**Conclusions:**

Our research has provided valuable insights into the role of TNFSF11, revealing its negative regulation of GPX4, which could be influential in crafting therapeutic strategies. These findings set the stage for further exploration into the mechanisms underpinning the relationship between TNFSF11 and GPX4, potentially opening up new avenues for precision medicine in the treatment of LUAD.

**Supplementary Information:**

The online version contains supplementary material available at 10.1186/s12967-024-05112-y.

## Background

Lung cancer is one of the most frequently diagnosed cancers and the leading cause of cancer-related deaths worldwide [1]. Despite advances in treatment modalities for diagnosis and prognosis, lung cancer remains a leading cause of death worldwide [2, 3]. According to the Surveillance, Epidemiology, and End Results Program (SEER) estimates, there will be an additional 236,740 new cases of lung cancer diagnosed in the U.S. in 2022, accounting for 12.3% of all new cancer cases. Furthermore, lung cancer is expected to cause 130,180 deaths in the same year. According to SEER data, the 5-year relative survival rate from 2012 to 2018 for lung cancer was 22.9%. More than half of the patients were diagnosed with distant metastasis, and their 5-year relative survival rate was less than 6% [4]. The incidence of lung cancer is relatively high in China, with approximately 406 million new cases and 240 million cancer-related deaths reported in 2016. Despite the incidence rates have remained stable in men, there has been a 2.3% annual increase in women from 2000 to 2016 [5]. Lung adenocarcinoma (LUAD) accounts for almost half of lung cancers and is a major histological type [6]. Although molecular targeted therapy and checkpoint immunotherapy have led to prolonged survival in some LUAD patients, many patients are still unable to benefit from these treatments [7–9]. The initial response to treatment may be promising, but drug resistance often develops in many patients, leading to disease progression and treatment failure. There is an urgent need for an effective therapeutic approach that can be tailored to the patient and tumor characteristics. Additionally, hyper-progressive disease (HPD) and immunotherapy-related adverse effects have hindered the clinical adoption of immunotherapy for certain patients [10–14]. Thus, exploring novel biomarkers to decipher the therapeutic strategies for LUAD patients is urgently required.

Tumor necrosis factor superfamily 11 (TNFSF11), also named as RANKL, TRANCE, ODF, and OPGL, is a type II homotrimeric transmembrane protein located mainly in the plasma membrane [15]. Besides the membrane-bound form (mRNAKL), it can also be cleaved by proteolysis from the cell membrane to yield a soluble form (sRANKL). Although both the soluble and the membrane-bound forms can bind to TNFRSF11A (RANK), It has been reported that the membrane-bound form of RANKL is the primary form involved in triggering the RANKL/RANK signaling pathway and mediating the generation of osteoclasts [16–19]; Besides its pivotal in bone remodeling, TNFSF11 has been revealed by scientists to play a critical role in the regulation of T cell-dependent immune responses and in mediating the interactions between T cells and dendritic cells [20, 21]. Recently, several studies have reported that TNFSF11 (namely RANKL) is closely related to breast cancer, gastric cancer, cervical, endometrial, prostate cancer, and cancer-related bone metastasis [22–26]. Jun Ah Lee reported osteosarcoma patients with high RANKL/TNFSF11 expression were less responsive to neoadjuvant chemotherapy, and RANKL overexpression was associated with shorter survival [27]. In another study, Gaoping Chen et al. found a relationship between RANKL expression and aggressive, advanced, metastatic prostate carcinoma [28]. A team led by Valerie A Odero-Marah established a close connection between RANKL and epithelial-mesenchymal transition (EMT) [29]. Li-Mien Chen et al. studied the role of the RANKL/RANK axis in human lung cancer (A549) cells, discovering its ability to activate ICAM-1 and enhance tumor migration, a process that could be inhibited by PDTC, a NF-κB pathway inhibitor [30]. Julien Faget reported a variation in the expression of RANKL between KRAS mutant and wild type lung cancer samples, with overexpression of RANKL being linked to poorer prognosis [31]. Despite this, the comprehensive expression pattern of TNFSF11 (RANKL) across various types of cancer and its relationship with clinicopathological parameters remains largely unexplored. Furthermore, the specific role of TNFSF11 in LUAD warrants additional investigation.

**Fig. 1:**
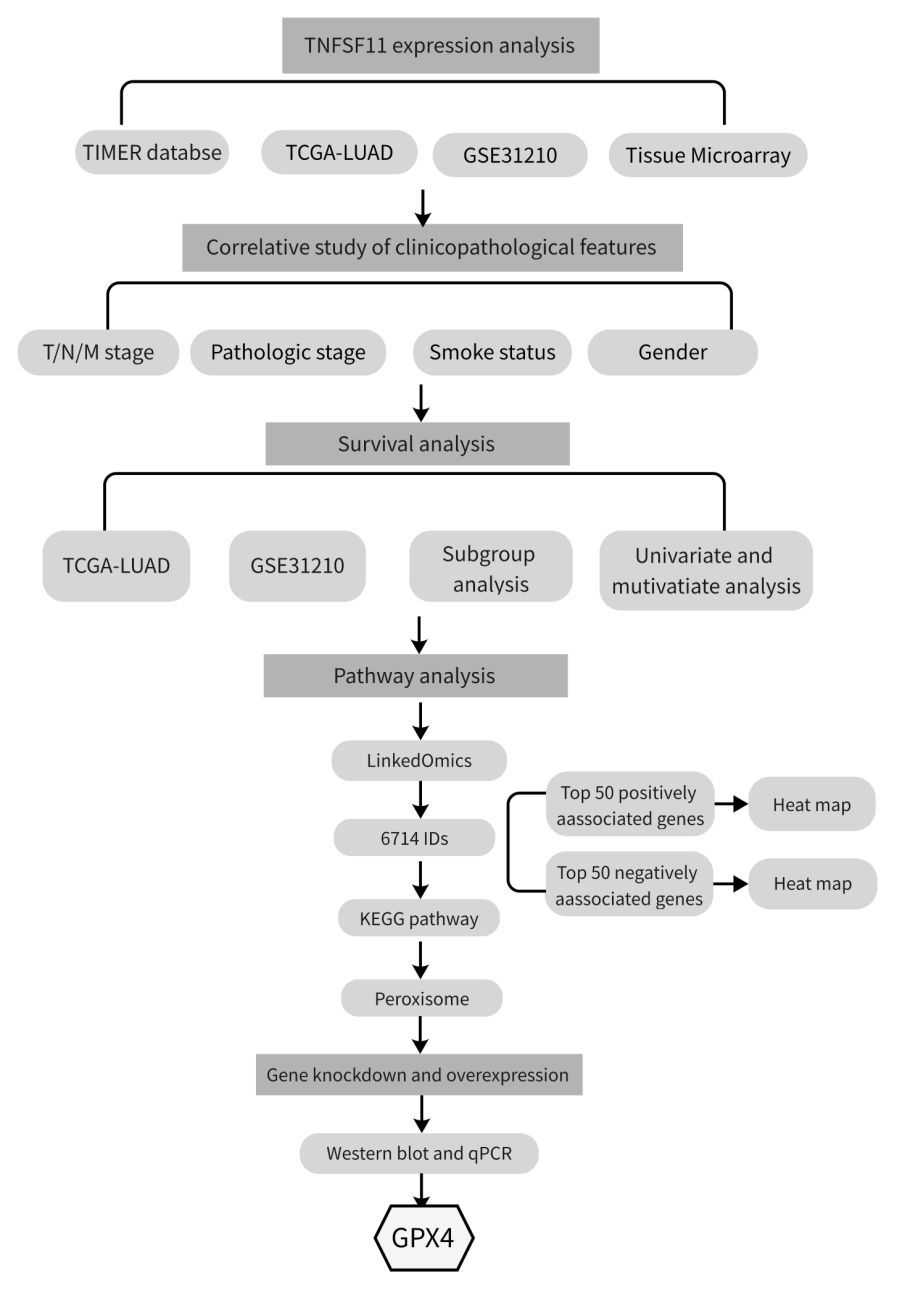
Flowchart of this study

In this study, we investigated TNFSF11 expression in LUAD using public databases and a tumor tissue microarray. The Gene Set Enrichment Analysis (GSEA) was employed to decipher the underlying roles of TNFSF11 in LUAD pathogenesis (Fig. 1). Further, in vitro experiments were performed to examine the relationship between TNFSF11 and peroxisome related genes within LUAD tumor cells. An immune infiltration analysis was also conducted to elucidate the association of TNFSF11 with immune infiltration. This investigation sheds light on the expression pattern of TNFSF11 in LUAD, offering a potential new therapeutic avenue in the treatment of LUAD.

## Materials and methods

### Data collection

The RNA-seq gene expression and clinical-pathological information were downloaded from the TCGA database (https://portal.gdc.cancer.gov/) in Level-3 HTSeq-FPKM format, which were then converted into transcripts per million reads (TPM). After filtering, 594 samples were selected for further analysis, including 59 normal tissues and 535 lung adenocarcinoma tissues.

The patients were divided into two groups based on the TNFSF11 expression level, with the top 50% defined as the TNFSF11 high group and the lower half as the TNFSF11 low group. The clinicopathological characteristics of LUAD patients were summarized in Table 1. Additionally, the GSE31210 dataset from the Gene Expression Omnibus (GEO) (http://www.ncbi.nlm.nih.gov/geo/) database was obtained and used as an external validation for TNFSF11 gene expression analysis.

**Table 1:** Clinical characteristics of the lung adenocarcinoma patients (TCGA)

Characteristic	Low expression of TNFSF11	High expression of TNFSF11	p
N	267	268	
T stage, n (%)			0.812
T1	92 (17.3%)	83 (15.6%)	
T2	139 (26.1%)	150 (28.2%)	
T3	25 (4.7%)	24 (4.5%)	
T4	10 (1.9%)	9 (1.7%)	
N stage, n (%)			0.053
N0	187 (36%)	161 (31%)	
N1	42 (8.1%)	53 (10.2%)	
N2	29 (5.6%)	45 (8.7%)	
N3	1 (0.2%)	1 (0.2%)	
M stage, n (%)			0.640
M0	177 (45.9%)	184 (47.7%)	
M1	14 (3.6%)	11 (2.8%)	
Pathologic stage, n (%)			0.080
Stage I	158 (30%)	136 (25.8%)	
Stage II	59 (11.2%)	64 (12.1%)	
Stage III	32 (6.1%)	52 (9.9%)	
Stage IV	14 (2.7%)	12 (2.3%)	
Smoker, n (%)			0.487
No	41 (7.9%)	34 (6.5%)	
Yes	221 (42.4%)	225 (43.2%)	
OS event, n (%)			0.187
Alive	179 (33.5%)	164 (30.7%)	
Dead	88 (16.4%)	104 (19.4%)	
Age, n (%)			0.929
<=65	129 (25%)	126 (24.4%)	
> 65	130 (25.2%)	131 (25.4%)	
Gender, n (%)			0.575
Female	139 (26%)	147 (27.5%)	
Male	128 (23.9%)	121 (22.6%)	
Age, meidan (IQR)	66 (59, 72)	66 (58, 72)	0.525

### Gene expression analysis

Tumor Immune Estimation Resource (TIMER) (https://cistrome.shinyapps.io/Timer), an online database for gene expression and immune infiltrates analysis, was used to analyze TNFSF11 expression in pan cancers. The ggplot2 package in R software was used to compare the relative TNFSF11 expression in TCGA and GSE21310 databases. A *p*-value of less than 0.05 was defined as statistically significant.

### Construction of the ROC curve

ROC curve analysis is a useful tool to evaluate the diagnostic performance of a biomarker. The area under the ROC curve (AUC) represents the overall diagnostic accuracy of the biomarker, with an AUC of 1 indicating perfect accuracy and an AUC of 0.5 indicating no better than chance accuracy.

The receiver operator characteristic (ROC) curve was generated using pROC (version 1.17.0.1) and visualized by ggplot2 in R language (version 3.6.3) to evaluate the diagnostic efficacy of TNFSF11 in LUAD.

### Tissue microarray and immunohistochemistry (IHC) staining

**Fig. 2:**
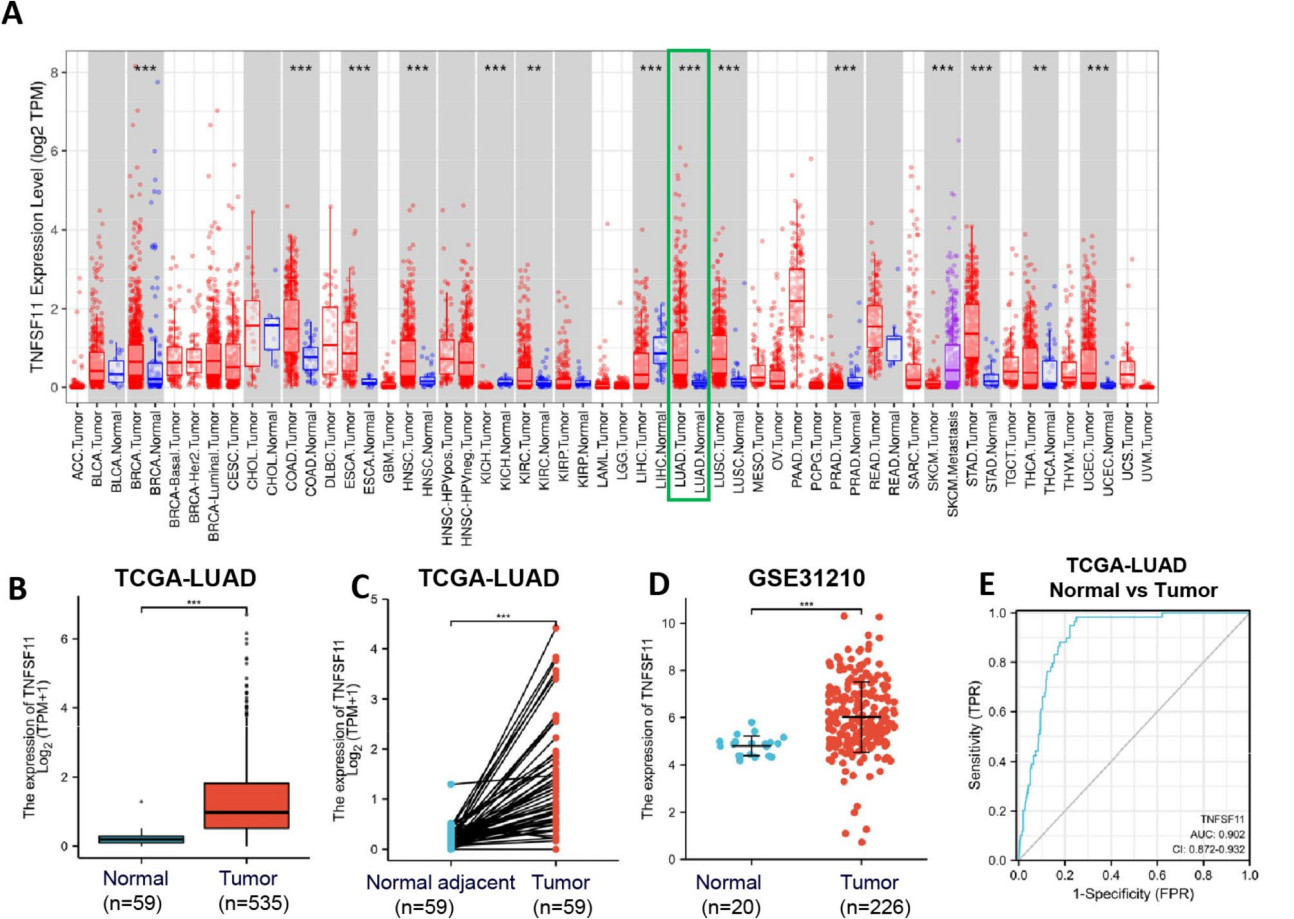
TNFSF11 mRNA expression in tumor and normal tissues. (**A**) The expression level of TNFSF11 in in different tumor types from TIMER database; (**B**) Expression levels of TNFSF11 in LUAD (*n* = 535) and normal tissue (*n* = 59) from TCGA database; (**C**) The expression of TNFSF11 in LUAD (*n* = 59) and its paired adjacent tissues (*n* = 59); (**D**) TNFSF11 mRNA expression in GSE31210 from GEO database; (**E**) Receiver operating characteristic analysis (ROC) of TNFSF11 in LUAD; (*P < 0.05, **P < 0.01, and ***P < 0.001)

Human LUAD tissue microarray (OD-CT-RsLug04-003) was obtained from Outdo Biotech Co. Ltd Shanghai, China. The array constitutes 54 human LUAD samples and 51 matched adjacent normal tissues (Fig. 2). The immunohistochemistry assay was performed as previously described (24). Briefly, the paraffin-embedded tissues were dewaxed, hydrated, and heated in an autoclave for antigen retrieval. The array was treated with methanol containing 3% H_2_O_2_ at room temperature. After washing and blocking with serum for 30 min, the array was incubated overnight at 4℃ with an anti-TNFSF11 (RANKL) antibody (Abcam, ab9957) at a concentration of 1:100. All slides were subsequently exposed to the secondary antibody on the second day. The ABC reagent was added and incubated for 30 min at room temperature. After washing with PBS, DAB was added and subsequently counterstained with hematoxylin. Finally, the section was dehydrated and mounted.

A staining index was calculated by multiplying the staining score and the percentage of positive cells. The intensity score was defined as 0 (no staining or negative staining), 1 (weak staining), 2 (moderate staining), and 3 (strong staining). To define the percentage of positive cells, negative cells were defined as 0, while the percentage of positive cells less than 10% were defined as 1. Cells with 10–50% positive were defined as 2, those with 51–80% positive were defined as 3, and those with greater than 80% positive were defined as 4. The data were presented as mean ± SEM.

For immunohistochemical analysis of mice tumor tissues, the procedure follows the previously outlined steps, employing the anti-4 Hydroxynonenal antibody (Abcam, ab48506) at a concentration of 1:100 for staining purposes.

### Kaplan Meier survival curve analysis

Survival and survminer packages were used to create Kaplan-Meier curves for comparative analysis of overall survival (OS) and progression-free survival (PFS) in TNFSF11 high and low patients.

### LinkedOmics gene association analysis and KEGG pathway enrichment analysis

**Fig. 3:**
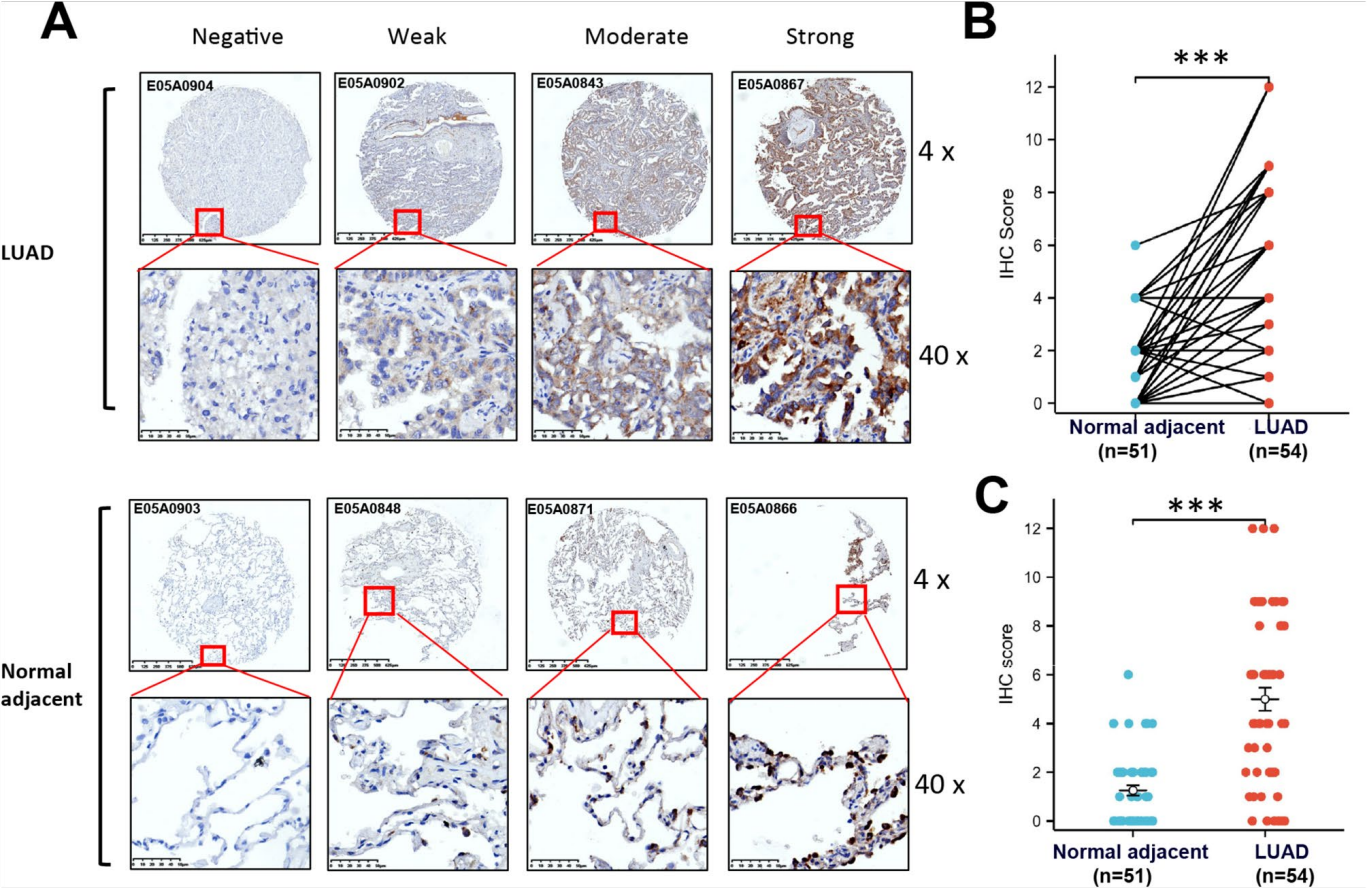
Immunohistochemical staining for TNFSF11 in LUAD and normal tissues. (**A**) Representative immunohistochemical staining for TNFSF11 expression in LUAD tissues and corresponding normal tissues by using tissue microarrays. The paired plots (**B**) and scatter plots (**C**) showing the immunohistochemical scoring of TNFSF11 expression in LUAD tissues (*n* = 54) and adjacent normal tissues (*n* = 51). (*** *P* < 0.001). Bar: mean ± SEM.

LinkedOmics (http://www.linkedomics.org/login.php) is a publicly available web server that integrates multi-dimensional omics data from TCGA for further analysis. In this study, LinkedOmics was used to identify the gene that is associated with TNFSF11 by using RNAseq dataset comprising 515 LUAD patients. The correlation analysis was conducted using the Spearman correlation test. Out of the 19,988 gene IDs analyzed, only 6714 were annotated within specific functional categories. These annotated genes were subsequently utilized for Kyoto Encyclopedia of Genes and Genomes (KEGG) pathway analysis, providing deeper insights into the biological pathways associated with TNFSF11 in lung adenocarcinoma. The top 50 positively and 50 negatively associated genes with TNFSF11were presented in heat maps (Fig. 3A).

### Cell culture

The human lung adenocarcinoma cell line NCI-H2126 was obtained from the Stem Cell Bank at the Chinese Academy of Sciences in Shanghai, China. The PC-9 cell line was provided by Dr. Gengpeng Lin from The First Affiliated Hospital at Sun Yat-Sen University in Guangzhou, China. Both PC-9 and H2126 cells were authenticated by STR profiling and maintained in RPMI 1640 medium supplemented with 10% FBS at 37 °C in 5% CO_2_.

### Lentivirus transduction

Two lentiviral vectors (LV3-H1/GFP&Puro) harboring shRNA targeted at human TNFSF11 were utilized to knockdown TNFSF11 expression. The sequences are as follows: shTNFSF11 #1: 5’-GGTCAGGGAATTCTGAATTCC-3’;shTNFSF11 #2: 5’-GGGCCAAGATCTCCAACATGA-3’;In addition, the LV5(EF-1a/ GFP&Puro) vector was used to overexpress TNFSF11. All these vectors and their respective non-targeted negative controls were acquired from Shanghai GenePharma Co. Ltd. (China).

The lentiviral transduction process was conducted in accordance with the manufacturer’s guidelines. In summary, 1 × 10^5^ cells were plated in a 24-well plate and incubated overnight at 37 °C within a humidified atmosphere containing 5% CO_2_. Post incubation, the cells were transduced with lentiviral vectors following the manufacturer’s instructions. Three days subsequent to transduction, the cells underwent puromycin selection. After this phase, the cells were harvested for additional experiments.

### Real time PCR

Total RNA was extracted from H2126 and PC-9 cells with TRIzol reagent (Life techologies #15596-026). cDNA was synthetized with 0.5–1 µg RNA by using a RT Master Mix for qPCR II (MCE, #HY-K0510A) and then mixed with SYBR-Green Master Mix for amplification by using the Bio Rad CFX96 system (Bio-Rad Laboratories). The relative mRNA expression was calculated and normalized withβ-actin.

The primers are shown as follows: TNFSF11-F: 5’-ATCGTTGGATCACAGCACATC-3’, TNFSF11-R: 5’-AGACTCACTTTATGGGAACCAGA-3’; GPX4-F: 5’-GAGGCAAGACCGAAGTAAACTAC-3’, GPX4-R: 5’- CCGAACTGGTTACACGGGAA-3’; β-actin-F: 5’-CATGTACGTTGCTATCCAGGC-3’; β-actin-R: 5’-CTCCTTAATGTCACGCACGAT-3’.

### Western blotting

Western blotting was performed as previously described [32, 33]. The following antibodies were used: anti-TNFSF11 (RANKL) (Abcam, ab9957); anti-Catalase (Cell Signaling Technology, 12,980); anti-GPX4 (Cell Signaling Technology, 52,455), anti-SOD1 (Proteintech, 67480-1-Ig); anti-4 Hydroxynonenal (Abcam, ab48506), anti-caspase 3 (Cell Signaling Technology, 9962), anti-caspase 9 (Cell Signaling Technology, 9502) and anti-α-Tubulin (Proteintech, 66031-1-Ig). Image J software was used for the quantitative analysis of each band, the relative protein expression was normalized with α-Tubulin.

### Cell proliferation assay

Cells were seeded in 96-well plates at a density of 5 × 10^3^ per well and incubated overnight, Subsequently, various concentrations of erastin (GLPBIO, GC16630), RSL3 (MCE, HY-100,218 A) and Ferrostatin-1 (GLPBIO, GC10380) were added into each well and incubated for 48–72 h, 10–20 µl of Cell Counting Kit-8 (CCK-8) were added to each well and incubated at 37ºC for 4 h. The absorbance was then measured at 450 nm.

### ROS detection

MitoSOX Red was obtained from MCE (HY-D1055), and used for superoxide detection. Briefly, cells were seeded in a plate and incubated overnight, the next day, erastin or/and ferrostatin were added in the 12 well-plates. After incubation for suitable times, cells were harvested and washed three times with PBS. Resuspend cells with PBS containing 1µM MitoSOX Red and incubated in the dark for 30 min at 37 °C, and immediately analyzed with flow cytometer (Beckman CytoFlex). Data were analyzed using CytExpert 2.4 software.

### Xenograft assay

Female BALB/c nude mice, aged 5–6 weeks, were acquired from Guangdong GermPharmatech Co., Ltd., Guangdong, China. They were maintained on a standard diet and water. All animal experiments were conducted with the approval and under the supervision of the Animal Care and Use Committee of Sun Yat-sen University (Approval #: L102012023080F).

For the experiment, 2 × 10^6^ PC-9 LV OE-NC cells or OE-TNFSF11 cells, suspended in 100 µl of PBS, were subcutaneously injected into the right flanks of the mice. The tumor volume was calculated according to the formula volume = 0.5 × length × width^2^. Seven days post-inoculation, the mice were divided randomly into vehicle and erastin treatment groups, with five mice in each group. Treatments were administered via intraperitoneal injection, with either vehicle or 20 mg/kg of erastin, once daily. Body weight and tumor volume were monitored weekly. On day 35, the mice were euthanized, and the tumors were excised, removed, and weighed.

### Hematoxylin and Eosin (HE) staining

HE staining was performed using the HE Staining Kit (Beyotime, C0105S) following the manufacturer’s guidelines. Initially, paraffin-embedded sections were deparaffinized in xylene, two cycles of 5 min each, to remove the paraffin wax. The sections were then rehydrated through a graded series of ethanol concentrations: starting with 100% ethyl alcohol for 5 min, followed by successive immersions in 90%, 80%, and 70% ethyl alcohol for 2 min at each concentration, and then rinsed in distilled water. Subsequently, sections were stained with hematoxylin solution for 5 min to specifically target and stain the nuclei. This was followed by a rinse under running tap water for 10 min to wash away excess stain. The differentiation process, which removes non-specific background staining, involved briefly dipping the sections in 1% acid alcohol for 10 s, followed by another rinse in running tap water for 10 min to stop the differentiation process. For counterstaining, which provides contrast by staining the cytoplasm and other tissue elements, sections were immersed in eosin solution for 30 s. This step was followed by a brief wash in running tap water for 5 min to remove any excess eosin. Dehydration of the sections was achieved by sequentially passing them through 70%, 80%, 90%, and 100% ethyl alcohol, spending 10 s at each concentration, to prepare the tissue for clearing. The clearing step, which makes the sections transparent, was performed in xylene with two cycles of 5 min each. Finally, the stained sections were mounted with a resinous medium to preserve them for microscopy examination.

### Statistical analysis

The Wilcoxon signed-rank test, paired-samples t-test, and the Wilcoxon rank-sum test were utilized for both paired and non-paired comparisons. The Kruskal-Wallis test in conjunction with the Wilcoxon rank-sum test served to analyze correlations between TNFSF11 gene expression and clinical parameters. The construction of survival curves was achieved through the Kaplan-Meier method, with the log-rank test applied for further analysis. Correlation analysis of genes was performed employing Spearman’s analysis. The univariate and multivariate Cox regression analyses were carried out to evaluate overall survival. All statistical analyses were conducted using the R software package (version 3.6.3) and the Statistical Package for Social Sciences (version 24.0). A p-value of less than 0.05 was considered indicative of statistical significance.

## Results

### TNFSF11 mRNA was highly expressed in LUAD patients

The TIMER analysis revealed that TNFSF11 was highly expressed in various tumor types, including breast carcinoma (BRCA), colon adenocarcinoma (COAD), esophageal carcinoma (ESCA), head-neck squamous cell carcinoma (HNSC), kidney chromophobe (KICH), liver hepatocellular carcinoma (LIHC), kidney renal clear cell carcinoma (KIRC), lung squamous cell carcinoma (LUSC), prostate adenocarcinoma (PRAD), skin cutaneous melanoma (SKCM), stomach adenocarcinoma (STAD), thyroid carcinoma (THCA), uterine corpus endometrial carcinoma (UCEC). Notably, this expression is particularly significant in lung adenocarcinoma (LUAD) (*P* < 0.001; Fig. 2A).

To confirm the TNFSF11 expression levels in LUAD patients, we analyzed the data downloaded from TCGA database. The findings revealed the substantial overexpression of TNFSF11 in LUAD samples than in normal tissues (*P* < 0.001; Fig. 2B). In addition, a pairwise comparison of 59 LUAD tissues with adjacent normal tissues confirmed the higher expression of TNFSF11 in the tumor tissues than the paired normal tissues (*p*<0.001; Fig. 2C). GSE31210 dataset analysis also confirmed the elevated TNFSF11 expression in tumor tissues than the normal lung tissues (*p*<0.001; Fig. 2D). However, the TNFSF11 mRNA expression level was not associated with T/N/M classification, pathologic stage, smoking status, or gender (Supplementary Fig. 1A-F).

In addition, a receiver operating characteristic (ROC) curve was drawn, and the area under the curve (AUC) of TNFSF11 was 0.902, suggesting TNFSF11 is a powerful diagnostic factor to differentiate tumors from normal tissue (Fig. 2E).

### TNFSF11 protein was overexpressed in LUAD tissues

Immunohistochemical staining was employed to validate the expression of TNFSF11 at the protein level on a tissue array. As depicted in Fig. 3A, the representative immunohistochemical staining results for normal and tumor tissues are provided, TNFSF11 is expressed in both normal tissues and tumor tissues, with localization on the cell membrane. The associated IHC scores for the tissues are presented in Fig. 3B, indicating a significantly higher expression of TNFSF11 in LUAD tissues as compared to adjacent normal tissues (*p* < 0.001). In Fig. 3C, a scatter plot meticulously illustrates the distribution of immunohistochemistry scores across various tissue groups. Specifically, in normal tissues, only 7 out of 51 specimens exhibited an immunohistochemistry score of 4 or higher. Conversely, within tumor tissues, a significant increase is observed, with 34 out of 54 specimens scoring 4 or above. These findings strongly indicate an elevated expression of TNFSF11 protein in lung adenocarcinoma (LUAD) tissues compared to adjacent normal tissues.

### High TNFSF11 gene expression predicts an adverse outcome in LUAD

**Fig. 4:**
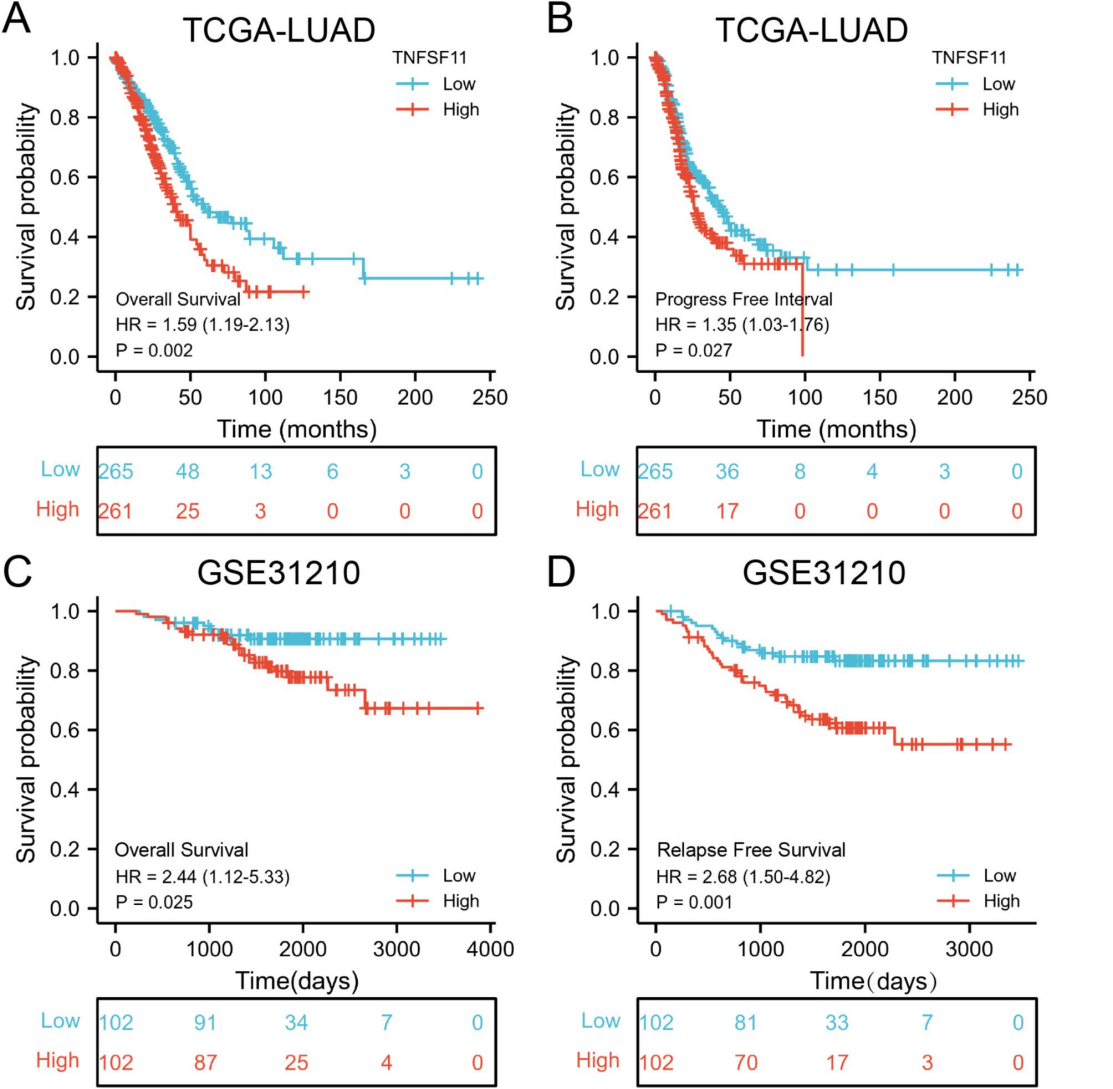
Survival analysis in patients with high and low TNFSF11 expression. (**A** and **B**) Survival curves for OS and PFI from TCGA data (*n* = 526); (**C** and **D**) Survival curves for OS and RFS from the GSE31210 dataset (*n* = 204)

Comprehensive clinical data is presented in Table 1. As illustrated in Fig. 4A-B, Kaplan-Meier analysis indicated that patients with high TNFSF11 expression had significantly shorter overall survival (OS) and progression-free intervals (PFI). Specifically, the median OS for the high TNFSF11 expression group was 40.3 months (range 33.3–54.1 m), which was markedly shorter than the low expression group (median OS 59.7 months; range 49.7–112), yielding a hazard ratio of 1.59 (95% CI: 1.19–2.13, *p* = 0.002; Fig. 4A). The median PFI in the TNFSF11 high group was 25.9 months (range 23.1–33.9), while it was 43.1 months in the low expression group (range 34.9–63.1, Fig. 4B). These results imply that elevated TNFSF11 expression correlates with unfavorable outcomes in lung adenocarcinoma patients. Our findings were corroborated by results obtained from the GSE31210 dataset (Fig. 4C-D). The high TNFSF11 expression group (*n* = 102) demonstrated significantly shorter overall survival (HR = 2.44, 95% CI: 1.12–5.33, *p* = 0.025) and relapse-free survival (RFS) (HR 2.68, 95% CI: 1.50–4.82, *P* = 0.001) compared to the low TNFSF11 expression group (*n* = 102). Subgroup analyses further illustrated that high TNFSF11 expression was significantly linked to shorter overall survival in patients who were younger than 65 years, female, and smokers. These results are presented in Supplementary Fig. 2.

Multivariate analyses were performed to identify independent predictors of survival. Our results indicated that the TNFSF11 expression level (HR = 1.866, 95% CI: 1.171–2.973, *p* = 0.009) stands as an independent prognostic factor for overall survival in LUAD. Other factors that were also identified as independent prognostic factors for overall survival included T stage (HR = 2.272, 95% CI: 1.119–4.614, *p* = 0.023), primary therapy outcome (HR = 0.369, 95% CI: 0.224–0.606, *p* < 0.001), and the presence of residual tumors (HR = 2.998, 95% CI: 1.183–7.579, *p* = 0.021). The details of these findings are presented in Table 2.

**Table 2:** Univariate and multivariate analysis of clinicopathological factors that correlate with OS of LUAD patients

Characteristics	Total(N)	Univariate analysis		Multivariate analysis	
		Hazard ratio (95% CI)	P value	Hazard ratio (95% CI)	P value
T stage	523				
T1&T2	457	Reference			
T3&T4	66	2.317 (1.591–3.375)	< 0.001	2.272 (1.119–4.614)	0.023
N stage	510				
N0	343	Reference			
N1&N2&N3	167	2.601 (1.944–3.480)	< 0.001	1.350 (0.817–2.233)	0.242
M stage	377				
M0	352	Reference			
M1	25	2.136 (1.248–3.653)	0.006	1.748 (0.663–4.607)	0.259
Gender	526				
Female	280	Reference			
Male	246	1.070 (0.803–1.426)	0.642		
Age	516				
<=65	255	Reference			
> 65	261	1.223 (0.916–1.635)	0.172		
Pathologic stage	518				
Stage I&Stage II	411	Reference			
Stage III&Stage IV	107	2.664 (1.960–3.621)	< 0.001	1.496 (0.771-2.900)	0.234
Primary therapy outcome	439				
PD&SD	108	Reference			
PR&CR	331	0.377 (0.268–0.530)	< 0.001	0.369 (0.224–0.606)	< 0.001
Residual tumor	363				
R0	347	Reference			
R1&R2	16	3.879 (2.169–6.936)	< 0.001	2.998 (1.183–7.597)	0.021
TNFSF11	526				
Low	265	Reference			
High	261	1.594 (1.191–2.133)	0.002	1.866 (1.171–2.973)	0.009

### Co-expression analysis of TNFSF11 and pathway investigation in LUAD

**Fig. 5:**
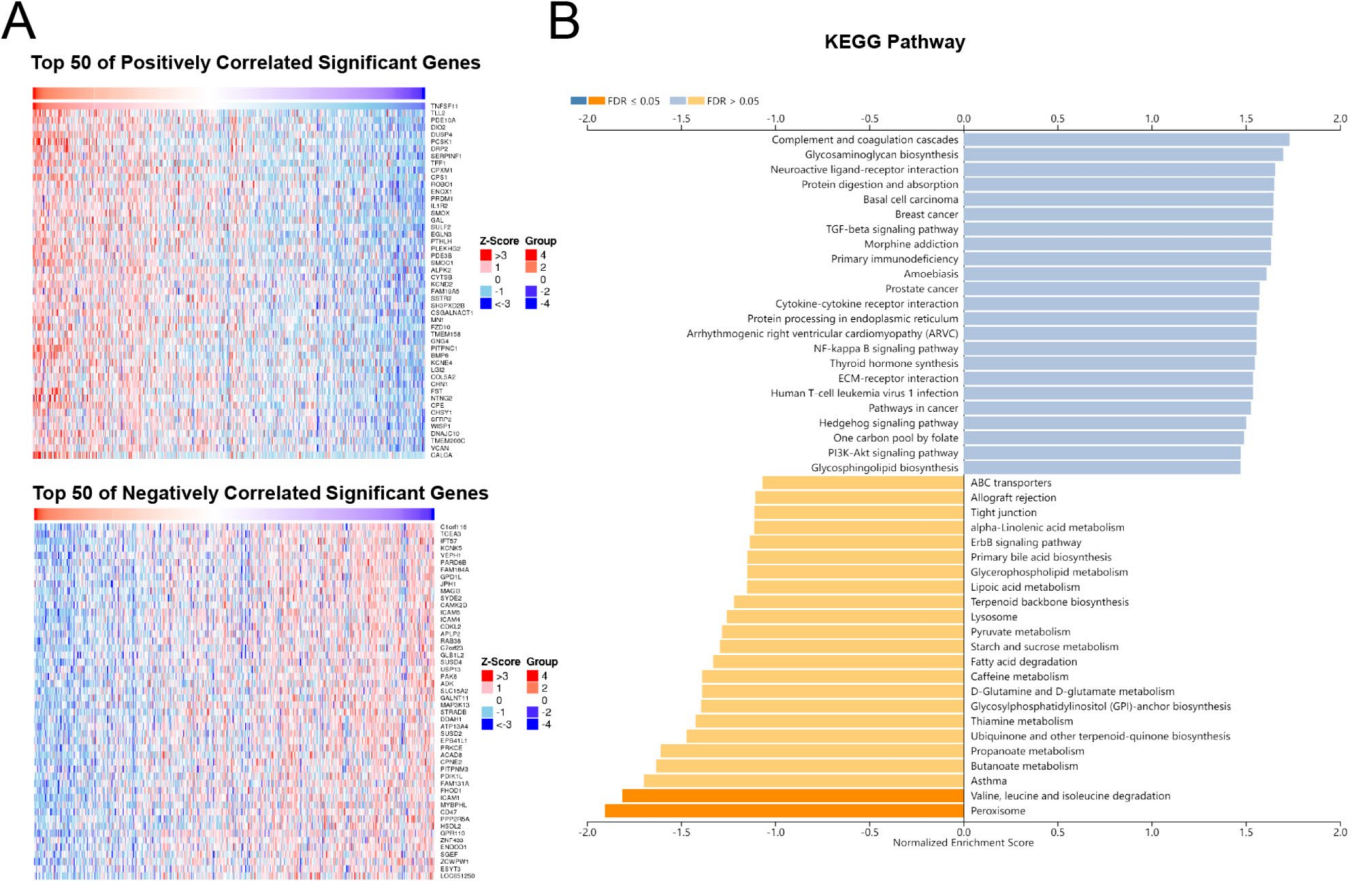
Gene co-expression analysis with TNFSF11 in LUAD (LinkedOmics). (**A**) 6714 genes were identified by LinkedOmics with the Spearman correlation test, the top 50 positively and 50 negatively associated genes are shown in a heat map. (**B**) All of the 6714 correlated genes were uploaded for Kyoto Encyclopedia of Genes and Genomes (KEGG) pathway analysis, the top 25 positively and 25 negatively associated pathways were shown

To investigate the underlying biological functions of TNFSF11 in LUAD, we utilized the online platform Linkedomics to analyze the genes significantly correlated with TNFSF11 in LUAD patients [34]. This approach resulted in the identification of 6714 genes significantly correlated with TNFSF11. A heatmap, depicted in Fig. 5A, showcases the top 100 of these genes, including 50 with positive correlation and 50 with negative correlation.

For a deeper insight into the functional implications, we performed a KEGG pathway analysis using the WEB-based Gene SeT AnaLysis Toolkit, incorporating all 6714 identified genes. This analysis highlighted that the degradation pathways of the branched-chain amino acids valine, leucine, and isoleucine, as well as peroxisome pathways, are significantly associated with the TNFSF11 gene (Fig. 5B), suggesting potential metabolic impacts and regulatory roles of TNFSF11 in LUAD.

### TNFSF11 expression was negatively associated with GPX4

**Fig. 6:**
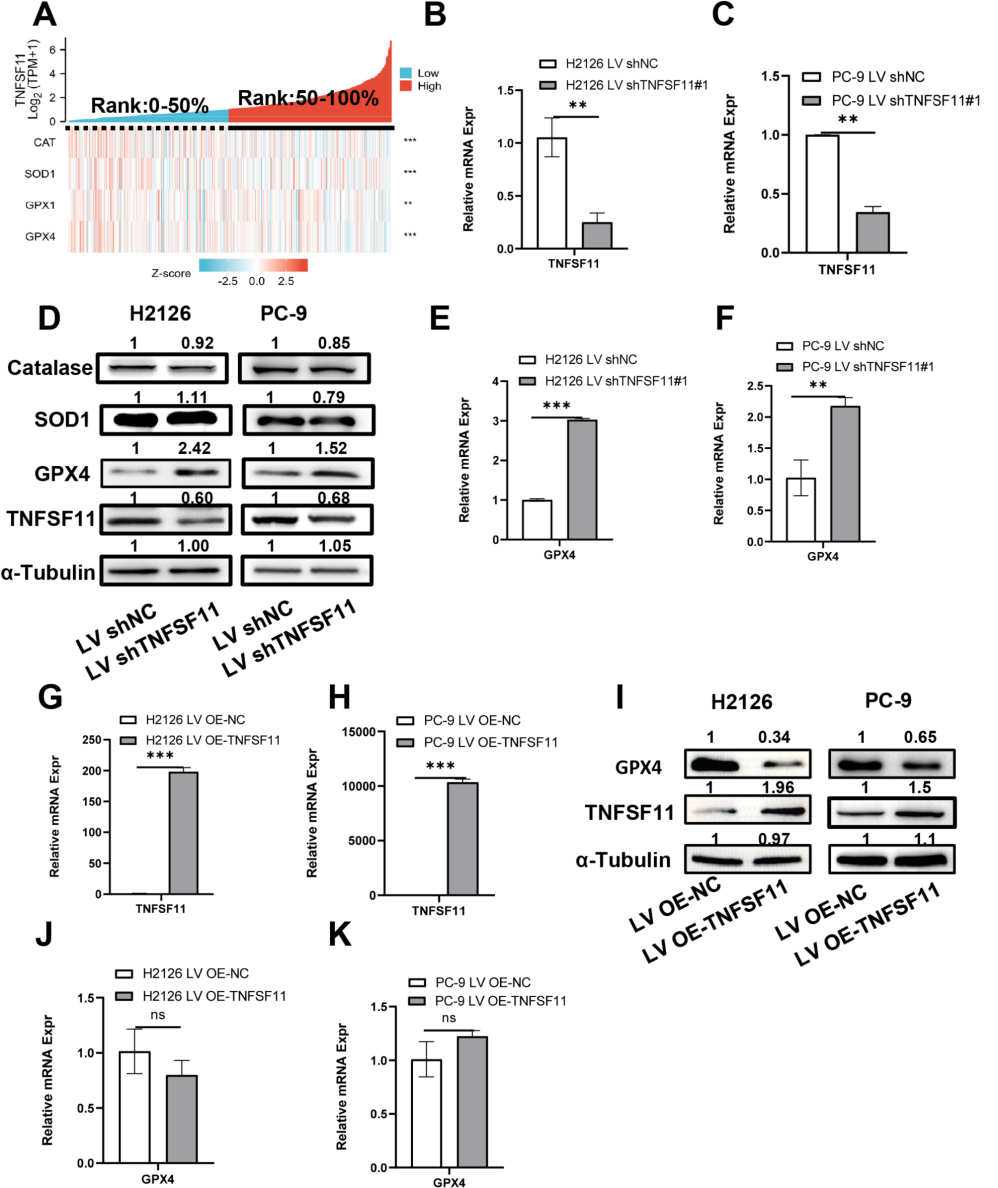
TNFSF11 expression is negatively correlated with GPX4. (**A**) Heat map illustrating the relationship between TNFSF11 expression and antioxidative enzymes, namely CAT, SOD1, GPX1, and GPX4 (**P < 0.01, and ***P < 0.001). (**B, C, E, F, G, H, J, K**) Comparison of mRNA expression of TNFSF11 and GPX4 post lentiviral transduction in H2126 and PC-9 cells. Bars, means ± SD, *n* = 3. **P < 0.01, ***P < 0.001, NS, no significant. (**D** and **I**) Western blot analysis of CAT, SOD1, GPX4, and TNFSF11 expression post lentiviral transduction in H2126 and PC-9 cells. α-Tubulin was used as a loading control. All results were repeated for three times. CAT: Catalase

The peroxisome, a ubiquitous membrane-bound organelle present in all eukaryotic cells, is instrumental in preserving cellular redox equilibrium. Peroxisomal dysfunction can trigger an imbalance in ROS, heightening levels of free radicals and, consequently, cellular oxidative stress [35, 36]. As outlined previously, we uncovered a substantial association between TNFSF11 and peroxisome-related genes. We, therefore, proceeded to scrutinize the relationship between TNFSF11 and several antioxidant enzymes, including Catalase (CAT), Superoxide Dismutase 1 (SOD1), Glutathione Peroxidase 1 (GPX1), and Glutathione Peroxidase 4 (GPX4). As depicted in Fig. 6A, our findings revealed that all these genes are negatively correlated with TNFSF11 expression. This indicates that TNFSF11 could exert an inhibitory influence on the gene expression of these antioxidant enzymes, potentially leading to an elevation in ROS and cellular oxidative stress.

To elucidate the relationship between TNFSF11 and antioxidant enzymes, we utilized shRNA to suppress TNFSF11 expression and subsequently performed Western blotting to probe the protein expression levels of antioxidative enzymes. Real time qPCR was conducted to verify the transduction efficiency and the GPX4 gene expression before and after lentivirus transductions. As depicted in Fig. 6B-F, we observed a significant upregulation of GPX4 at both gene and protein level when TNFSF11 was inhibited in H2126 and PC-9 cells. Conversely, the expression levels of CAT and SOD1 were not substantially affected. Additionally, we transduced PC-9 and H2126 cells with LV5-TNFSF11 to establish TNFSF11-overexpressing cell lines. As demonstrated in Fig. 6G-K, we observed a decrease of GPX4 protein expression in PC-9 and H2126 LV OE-TNFSF11 cells, however the mRNA of GPX4 was not significantly affected. Collectively, these findings suggest that TNFSF11 negatively regulates GPX4 expression in protein level, but in mRNA level. These results suggest that TNFSF11 may not be directly involved in the genetic regulation of GPX4.

### Cells overexpressing TNFSF11 are more susceptible to ferroptosis inducers

**Fig. 7:**
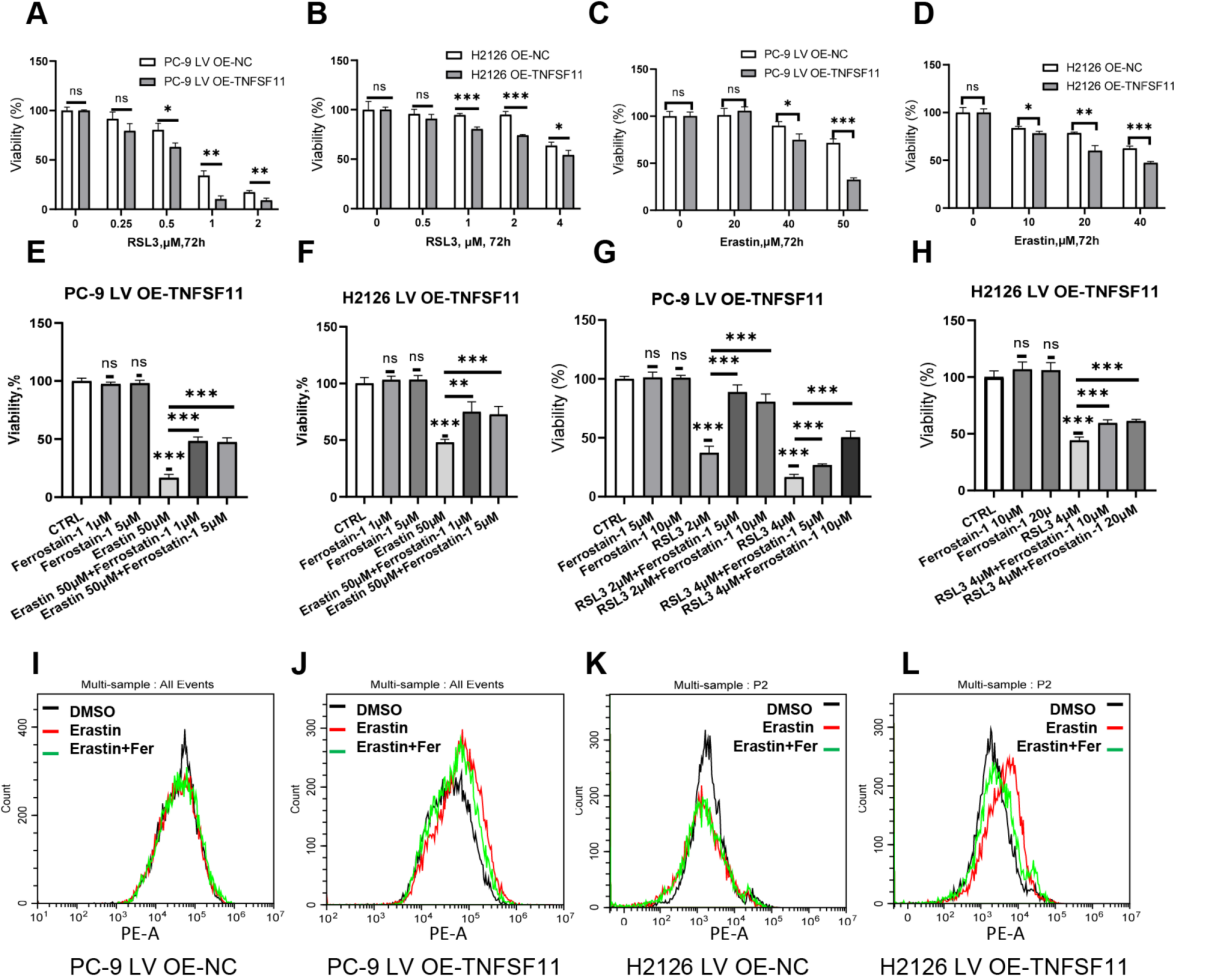
TNFSF11 overexpressing cells are more susceptible to ferroptosis inducers. (**A-D**) Cells were treated with various concentrations of RSL3 and erastin for 72 h and cell viability was measured using CCK8 assays. Statistical significance levels were shown above the curves. Bars, means ± SD, *n* = 3. *P < 0.05, **P < 0.01, ***P < 0.001, NS: no significant. (**E-H**) Cells were treated with RSL3, erastin or ferrostatin-1 for 24 h or 48 h, and subjected to CCK8 assay. Bars, means ± SD, *n* = 5. **P < 0.01, ***P < 0.001, NS: non-significant. (**I-L**) Intracellular ROS levels were evaluated using flow cytometry in cells post treatment with DMSO, erastin, or co-incubation with erastin and ferrostatin-1 for 4 h. MitoSox Red is used as a dye to detect ROS. All results were repeated for three times. Fer: Ferrostatin-1

GPX4 acts as a pivotal regulator of ferroptosis, a unique iron-dependent, non-apoptotic form of cell death. This regulation plays a crucial role in several cellular processes, including cell aging, oncogenesis, and cell death. When GPX4 is inhibited, lipid peroxidation ensues, leading to the onset of ferroptosis. Consequently, cells with elevated levels of GPX4 exhibit resistance to ferroptosis. In contrast, cancers with low GPX4 expression might be more susceptible to ferroptosis inducers [37]. To substantiate this hypothesis, we treated both LV OE-NC and LV OE-TNFSF11 cells with varying concentrations of erastin and RSL3, both of which are ferroptosis inducers. Our results showed that cells overexpressing TNFSF11 were more sensitive to these inducers, which is likely attributable to their diminished GPX4 expression levels (Fig. 7A-D). Remarkably, the inhibitory effect on cell growth imposed by these inducers could be counteracted by ferrostatin-1, a known inhibitor of ferroptosis (Fig. 7E-H). These observations suggest that ferroptosis inducers could offer a promising therapeutic avenue for targeting TNFSF11-expressing cancers. ROS detection experiments further demonstrated erastin could induce ROS elevation in the TNFSF11 overexpressing cells in 4 h, and this ROS production could be inhibited by ferrostatin-1 (Fig. 7I-L).

### Erastin significantly inhibited tumorigenesis in mice with high TNFSF11 expression

**Fig. 8:**
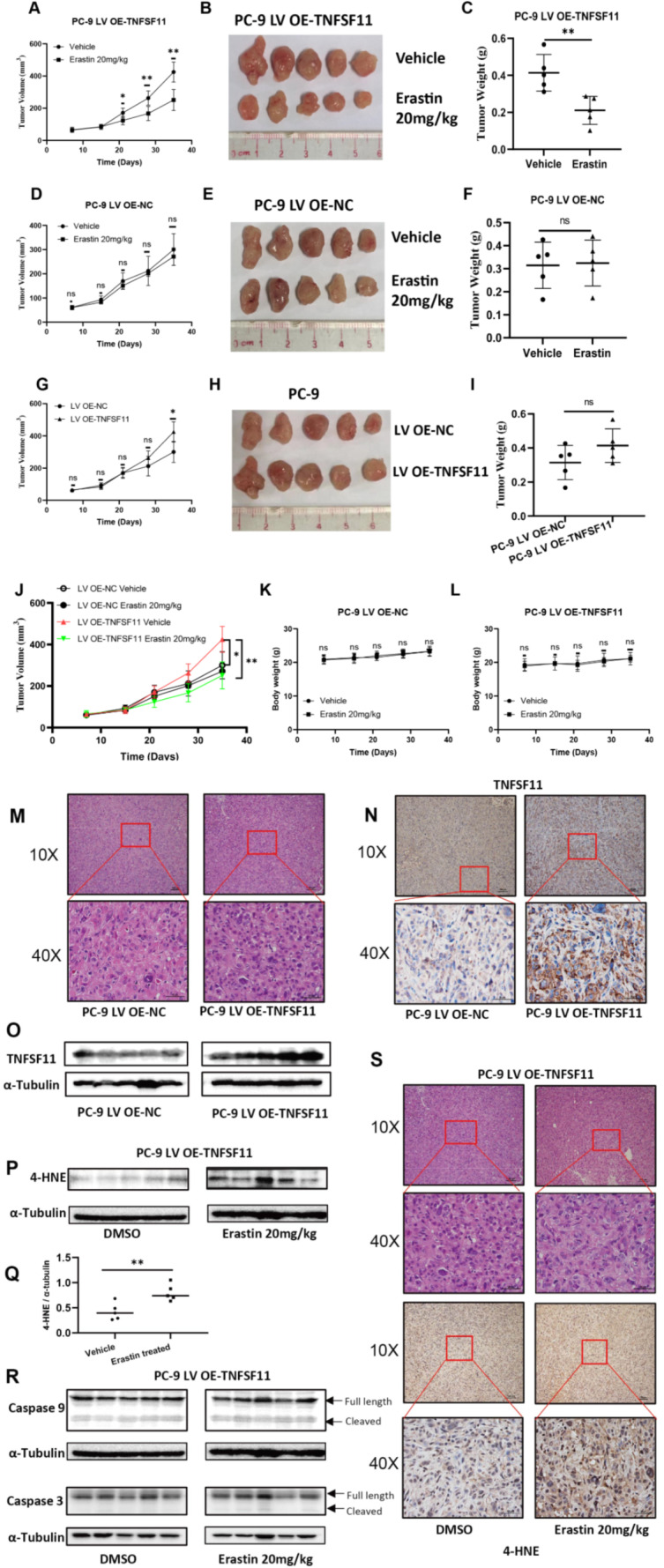
Antitumor activity of Erastin on xenograft models. PC-9 LV OE-TNFSF11 and OE-NC cells were injected subcutaneously into the right flank of nude mice. Seven days after inoculation, mice received daily intraperitoneal Erastin (20 mg/kg) or vehicle control. (**A**, **D**, **G**, **J**) The tumor growth curves for mice in each treatment group. (**B**, **E**, **H**) Images of isolated tumors from nude mice at the endpoint. (**C**, **F**, **I**) Tumor weights in different groups. (**K**, **L**) Body weight of nude mice in each group during the treatment period. (**M**) Representative Hematoxylin and Eosin (HE) staining images of PC-9 LV OE-NC and PC-9 LV OE-TNFSF11 tumors. (**N**) Representative Immunohistochemistry images display the expression of TNFSF11 in PC-9 LV OE-NC and PC-9 LV OE-TNFSF11 tumors. (**O**, **P**, **R**) Western blot analysis of 4-HNE, caspase 3, caspase 9 in PC-9 LV OE-TNFSF11 tumors treated with DMSO or Erastin, α-Tubulin was used as a loading control. All results were repeated for three times. (**Q**) Relative 4-HNE expression levels between DMSO-treated and Erastin-treated PC-9 LV OE-TNFSF11 tumor groups, normalized to tubulin, Bars, means ± SD, *n* = 5. **P < 0.01. (**S**) Representative HE staining and Immunohistochemistry images of PC-9 LV OE-TNFSF11 tumors stained with 4-Hydroxynonenal (4-HNE) following treatment with DMSO or Erastin. 4-HNE: 4-Hydroxynonenal

To further evaluate the therapeutic efficacy of ferroptosis inducers in TNFSF11-overexpressing tumors, we developed a subcutaneous lung cancer tumor model in mice using cells with TNFSF11 overexpression and their corresponding OE-NC counterparts. Both groups of mice, those with TNFSF11 overexpression and their corresponding NC counterparts, were treated with either a vehicle control or 20 mg/kg of erastin for 28 days. Our findings revealed a significant reduction in both the tumor growth rate and volume in the PC-9 LV OE-TNFSF11 mice compared to those treated with erastin (Fig. 8A and B). Notably, the final tumor weight was substantially lower in the erastin-treated group than in the vehicle group (Fig. 8C), underscoring erastin’s potent inhibitory impact on the growth of TNFSF11-overexpressing tumors. In contrast, erastin treatment to the PC-9 LV OE-NC tumor-bearing mice did not result in a significant alteration of the tumor growth rate, volume, or weight (Fig. 8D, E, F). Additionally, we noted that the tumor volume in the TNFSF11-overexpressing mice was significantly larger than that in the OE-NC group (Fig. 8G). While the average tumor weight was higher in the OE-TNFSF11 group compared to the OE-NC group, this difference in tumor weight was not statistically significant (Fig. 8H and I). As shown in Fig. 8J, we display the growth curves of four groups in a single chart for a more direct comparison of the tumor growth among the groups. From the chart, it is evident that the tumor growth volume in the OE-TNFSF11 group, after treatment with Erastin, is significantly inhibited, with the tumor volume being smaller than that of the OE-NC group. Importantly, there were no observed differences in body weight between the erastin-treated mice and the controls, indicating that erastin treatment is associated with favorable safety profiles (Fig. 8K and L).

The tumor tissues isolated from mice were analyzed using HE staining and Western blotting experiments. Representative images of HE staining are presented in Fig. 8M and S. Further, immunohistochemistry and Western blot experiments were conducted to ascertain the expression levels of TNFSF11 in PC-9 LV OE-NC and PC-9 LV OE-TNFSF11 tumors. The results indicated a significant overexpression of TNFSF11 in the PC-9 LV OE-TNFSF11 tumors, confirming the successful construction of the tumor model (Fig. 8N and O). Additionally, through Western blot analysis, we observed an increased expression of 4-Hydroxynonenal (4-HNE), a marker of lipid peroxidation and ferroptosis, in Erastin-treated PC-9 LV OE-TNFSF11 cells (Fig. 8P, Q, S), whereas there was no significant rise in the expression of apoptosis-related proteins, such as cleaved caspase 3 and cleaved caspase 9 (Fig. 8R). This confirms that the tumor growth inhibition effect induced by Erastin in PC-9 LV OE-TNFSF11 tumors is a result of ferroptosis, not apoptosis.

### Relationship between TNFSF11 expression and immune cell infiltration

**Fig. 9:**
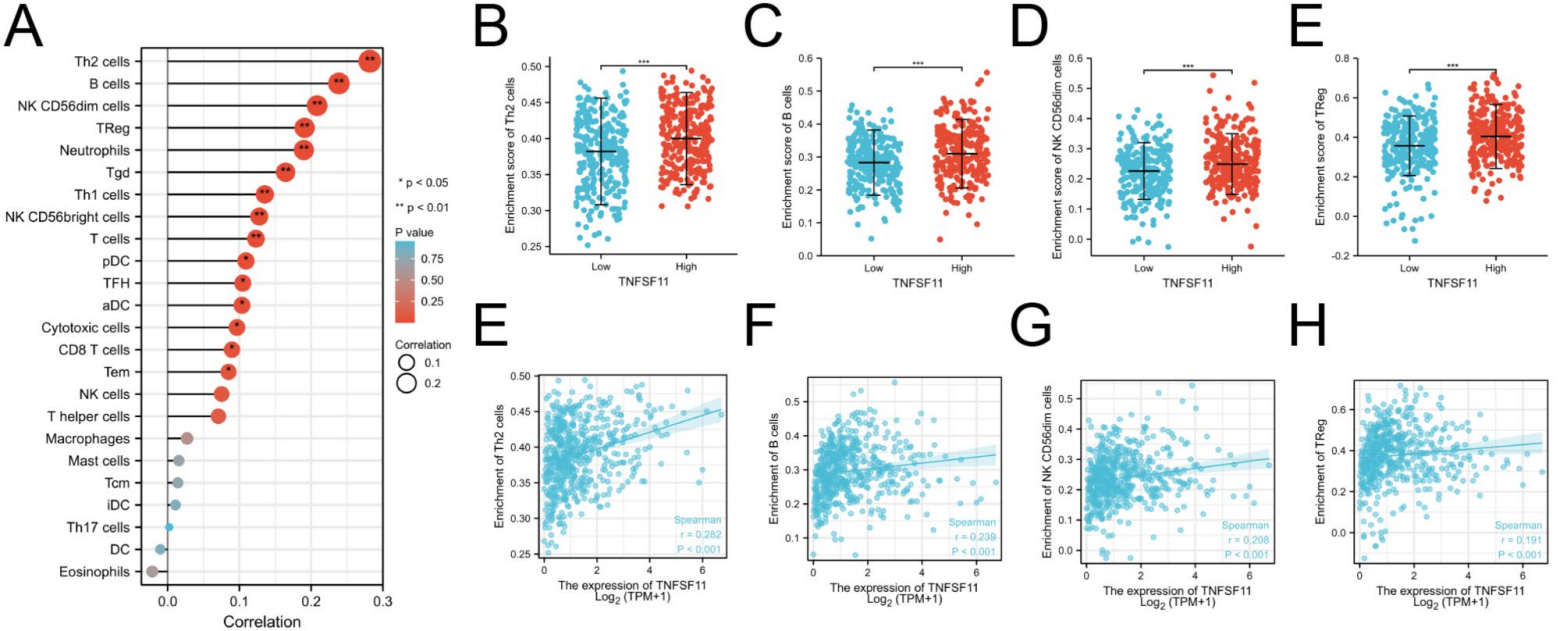
The correlation of TNFSF11 expression with immune infiltration level in LUAD. (**A**) Correlation analysis between TNFSF11 expression and immune cells. (**B–E**) The different infiltration levels of immune cells in TNFSF11 high and low groups. (**F–I**) Correlation diagrams to show the correlations between TNFSF11 and immune cells, *r* indicates *Spearman’s rho*. *P < 0.05; **P < 0.01; ***P < 0.001; ns: no significant

Based on previous reports, TNFSF11 has been associated with immune function. Therefore, in this study, we also investigated the relationship between TNFSF11 gene expression and immune cell enrichment using single-sample gene set enrichment analysis (ssGSEA). The results showed a positive correlation between TNFSF11 expression and type-2 T helper cells (Th2 cells) (*R* = 0.282, *p* < 0.001, Fig. 9A and E), B cells (*R* = 0.239, *p* < 0.001, Fig. 9A and F), NK CD56dim cells (*R* = 0.208, *p* < 0.001, Fig. 9A and G), treg cells (regulatory T cells) (*R* = 0.191, *p* < 0.001, Fig. 9A and H), but not correlated with eosinophins, dendritic cells (DC), type-17 T helper cells (Th17 cells), immature dendritic cells (iDC), central memory T cells (Tcm), mast cell and macrophages (Fig. 9A). Moreover, the relative infiltration analysis revealed a high level of Th2 cells, B cells, NK CD56dim cells, and Treg cells in the TNFSF11-high expression patients (Fig. 9B-E).

## Discussion

TNFSF11, initially recognized as a crucial regulator of bone metabolism via the RANK/RANKL/OPG pathway, has been increasingly implicated in the progression of various cancers, as well as in their tendency to undergo epithelial-mesenchymal transition [29, 38]. While its correlation with poor prognosis in LUAD has been documented [31], there has been no study thus far exploring its connection with clinical-pathological features or elucidating the intrinsic mechanisms through which it modulates tumor growth. Our study not only reveals that TNFSF11 is significantly overexpressed in LUAD patients relative to adjacent normal tissue but also discovers that such elevated expression is intricately associated with adverse clinical-pathological features, in the meanwhile, the relationship between TNFSF11 and immune infiltration was investigated. Most importantly, our research firstly unveiled that TNFSF11 plays a role in regulating cell survival through influence GPX4 expression, demonstrating a novel interaction where TNFSF11 negatively regulates GPX4 expression levels.

**Fig. 10:**
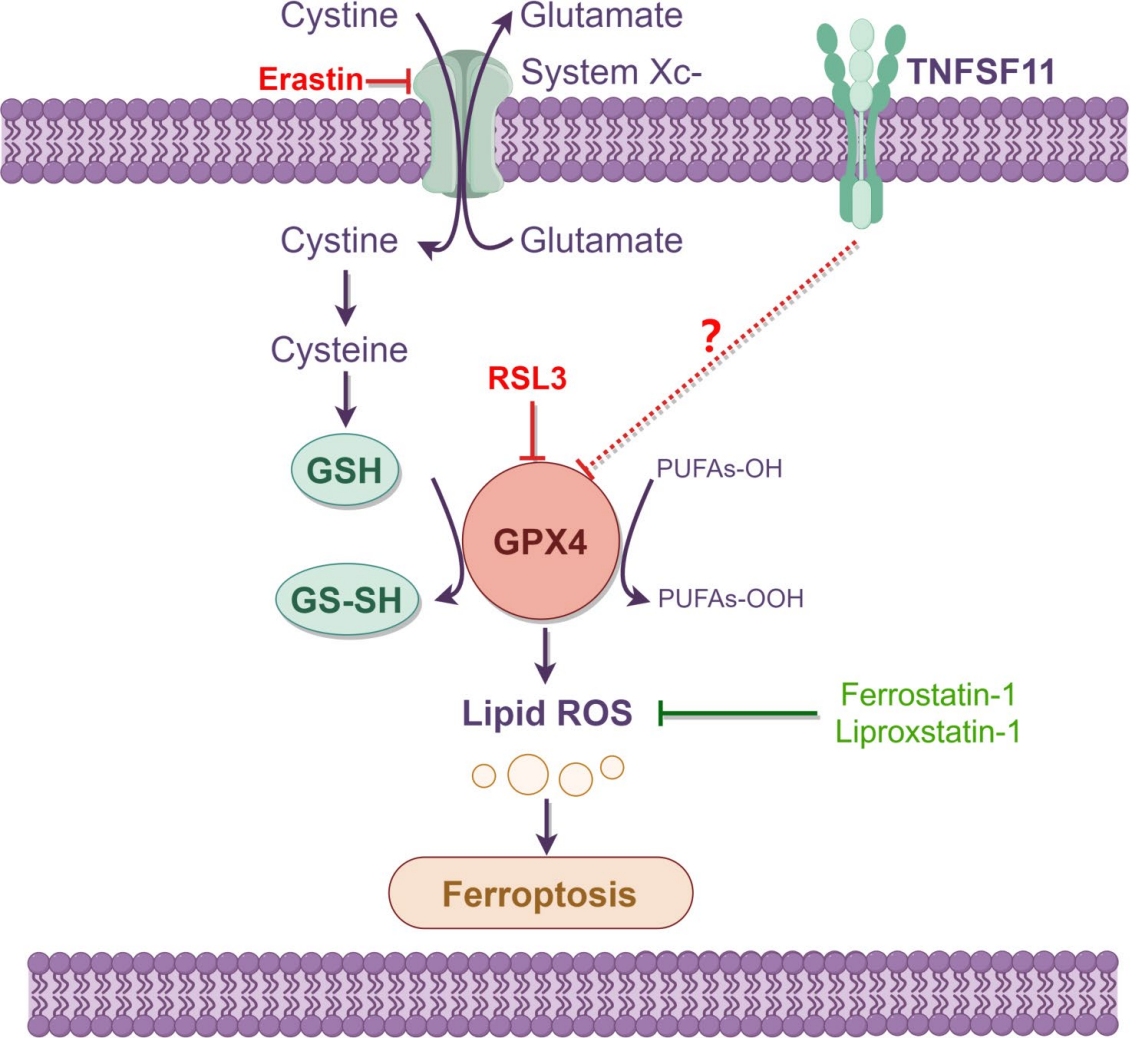
Schematic diagram to show the relationship between TNFSF11 and ferroptosis. TNFSF11 overexpression leads to reduced GPX4 expression, thereby increasing cellular susceptibility to ferroptosis inducers such as ferrostatin-1 and erastin. In contrast, cells exhibiting low TNFSF11 expression coupled with high GPX4 levels demonstrate enhanced resistance to these inducers of ferroptosis

Characterized by its reliance on iron and the accumulation of lipid reactive oxygen species (ROS), ferroptosis stands out as a regulated cell death mechanism distinct from apoptosis, necrosis, or autophagy. GPX4, as a pivotal actor in this process, assists tumor cells in mitigating lipid peroxidation, thereby fending off ferroptosis. As such, tumors with diminished GPX4 activity or expression potentially present as prime targets for ferroptosis inducers [39]. Cells expressing elevated levels of TNFSF11, given their tendency to harbor lower GPX4 expression, exhibit heightened susceptibility to ferroptosis inducers. As illustrated in Fig. 10, our findings reveal that TNFSF11 is markedly overexpressed in LUAD tissues, leading to reduced GPX4 expression. This diminished GPX4 expression heightens cellular vulnerability to ferroptosis inducers, including RSL3 and erastin. Conversely, cells with lower TNFSF11 expression, accompanied by elevated GPX4 levels, show increased resilience against these ferroptosis triggers. This concept opens up novel therapeutic avenues for lung cancer treatment.

As precision medicine comes to the forefront, stratifying lung cancer patients based on TNFSF11 expression becomes instrumental in tailoring therapeutic interventions. For those manifesting high TNFSF11 expression and a consequent bleak prognosis, agent inducing ferroptosis may emerge as a therapeutic mainstay. Our data accentuates the imperative of aligning cancer therapies with individual genetic landscapes, propelling precision oncology forward.

Although our research has uncovered a negative correlation between TNFSF11 and GPX4 at the protein level, and proposed the use of ferroptosis inhibitors for the treatment of TNFSF11 overexpressing cancers, the detailed regulatory mechanisms between TNFSF11 and GPX4 remain unclear and warrants further investigation. In our study, we observed an inverse expression relationship between TNFSF11 and GPX4. Notably, when TNFSF11 was knocked out, there was an increase in both the protein and mRNA levels of GPX4. This suggests that TNFSF11 negatively regulates GPX4 expression, at least in part. However, the relationship appears to be more complex than a simple inverse correlation. When TNFSF11 was overexpressed, there was no corresponding decrease in GPX4 mRNA levels, but there was a reduction in GPX4 protein levels. This discrepancy between mRNA and protein levels indicates that TNFSF11’s regulatory effect on GPX4 might occur post-transcriptionally. It’s possible that TNFSF11 influences GPX4 through a mechanism that affects protein stability or degradation rather than directly impacting mRNA synthesis or stability. This finding opens up new avenues for exploring the regulatory mechanisms of TNFSF11 and its potential role in cellular processes where GPX4 is a key player. Furthermore, we ascertained the pivotal immunomodulatory role of TNFSF11, consistent with previous literature [40, 41]. The intricate mechanisms and pathways wherein TNFSF11 modulate immune cell dynamics remain uncharted in our study and merit in-depth exploration. Such endeavors could shed light on innovative immunotherapeutic strategies. Anticipating synergy between immunotherapy and other therapeutic modalities, like targeted therapies or ferroptosis inducers, presents an exciting frontier in oncological interventions.

## Conclusions

In conclusion, our study demonstrated the adverse prognostic role of TNFSF11 in lung cancer, and also discovered a negative correlation between TNFSF11 and GPX4. Most importantly, we proposed the use of ferroptosis inhibitors to specifically target cells with high TNFSF11 expression as a treatment method for lung cancer. Our study may pave the way for innovative and precision medicine approaches in the treatment of lung adenocarcinoma (LUAD), based on differentiating levels of TNFSF11 expression.

### Electronic supplementary material

Below is the link to the electronic supplementary material.

**Supplementary Material 1: Supplementary Fig. 1**. The relationship between clinicopathological features and TNFSF11 mRNA expression. (**A**-**C**) The association of TNFSF11 expression and T/N/M classification in LUAD; (**D**) The association of TNFSF11 expression and pathologic stages; (**E**) The relationship between TNFSF11 expression and smoking status in LUAD patients; (**F**) The TNFSF11 expression in male and female; ns, no significance.

**Supplementary Material 2: Supplementary Fig. 2**. Kaplan-Meier survival curves for the patients, (**A** and **C**) under the age of 65 years old and over 65 years old, (**B** and **D**) male and female, (**C** and **E**) Different smoking status.

**Supplementary Material 3: Supplementary Fig. 3**. TNFSF11 expression levels are correlated with GPX4. (A) Comparison of mRNA expression of TNFSF11 and GPX4 post lentiviral transduction with LV shTNFSF11 #2 in H2126 cells. Bars, means ± SD, *n* = 3. *P < 0.05. (B) Western blotting analysis of GPX4 and TNFSF11 expression in protein level following lentiviral transduction with LV shTNFSF11 #2 in H2126 and PC-9 cells. α-Tubulin served as the loading control. Each experiment was conducted in triplicate.

## Data Availability

The datasets and materials of this study are derived from the following public databases. NCBI Gene Expression Omnibus (GEO) database (https://www.ncbi.nlm.nih.gov/geo/), The Cancer Genome Atlas (TCGA) database (https://portal.gdc.cancer.gov/), TIMER2.0 database (http://timer.comp-genomics.org/), LinkedOmics (http://www.linkedomics.org/login.php). Other data will be obtained from the first author, Amin Huang, upon reasonable request.

## Abbreviations

LUAD: lung adenocarcinoma
SEER: Surveillance, Epidemiology, and End Results Program
TNFSF11/RANKL: Tumor necrosis factor superfamily 11
HPD: Hyper-progressive disease
TPM: Transcripts per million reads
AUC: The area under the ROC curve
GEO: Gene Expression Omnibus
GSEA: Gene Set Enrichment Analysis
TIMER: Tumor Immune Estimation Resource
ROC: The receiver operator characteristic
OS: Overall survival
PFS: Progression-free survival
RFS: Relapse-free survival
PFI: progression-free intervals
KEGG: Kyoto Encyclopedia of Genes and Genomes
IHC: Immunohistochemistry
BRCA: breast carcinoma
COAD: colon adenocarcinoma
ESCA: esophageal carcinoma
HNSC: head-neck squamous cell carcinoma
KICH: kidney chromophobe
LIHC: Liver hepatocellular carcinoma
KIRC: kidney renal clear cell carcinoma
LUSC: lung squamous cell carcinoma
PRAD: prostate adenocarcinoma
SKCM: skin cutaneous melanoma
STAD: stomach adenocarcinoma
THCA: thyroid carcinoma
UCEC: uterine corpus endometrial carcinoma
CAT: Catalase
SOD1: Superoxide Dismutase 1
GPX1: Glutathione Peroxidase 1
GPX4: Glutathione Peroxidase 4
ssGSEA: single-sample gene set enrichment analysis
DC: dendritic cells
Th17 cells: type-17 T helper cells
iDC: immature dendritic cells
Tcm: central memory T cells
