# Supplementary figures and images for: Overexpression of TNFSF11 reduces GPX4 levels and increases sensitivity to ferroptosis inducers in lung adenocarcinoma
Source: J Transl Med. 2024 Apr 9;22:340. doi: 10.1186/s12967-024-05112-y (PMC11005202; doi:10.1186/s12967-024-05112-y)

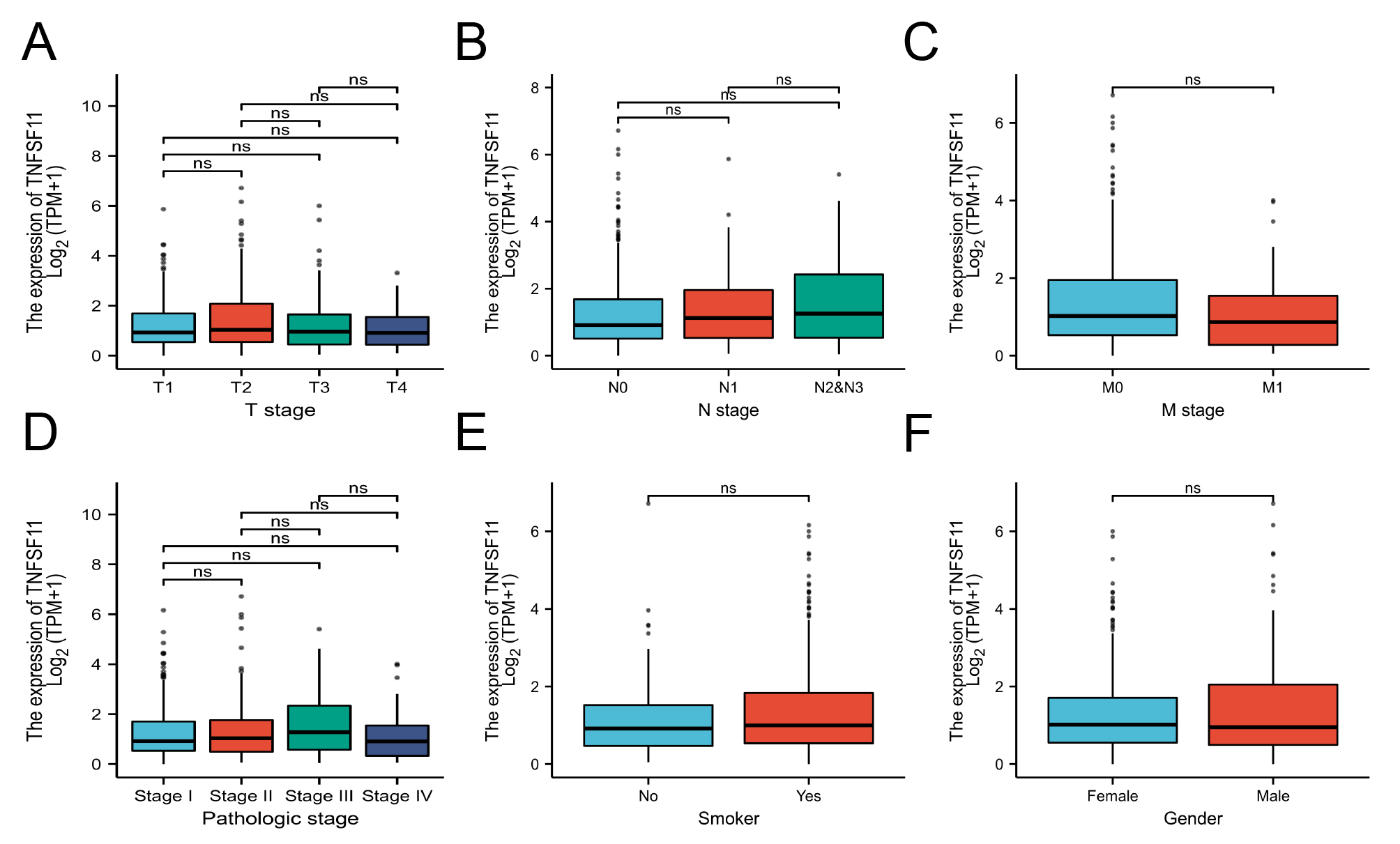

Supplement: Supplementary file 1 — Supplementary Material 1: Supplementary Fig. 1. The relationship between clinicopathological features and TNFSF11 mRNA expression. (A-C) The association of TNFSF11 expression and T/N/M classification in LUAD; (D) The association of TNFSF11 expression and pathologic stages; (E) The relationship between TNFSF11 expression and smoking status in LUAD patients; (F) The TNFSF11 expression in male and female; ns, no significance. [file 12967_2024_5112_MOESM1_ESM.tif]

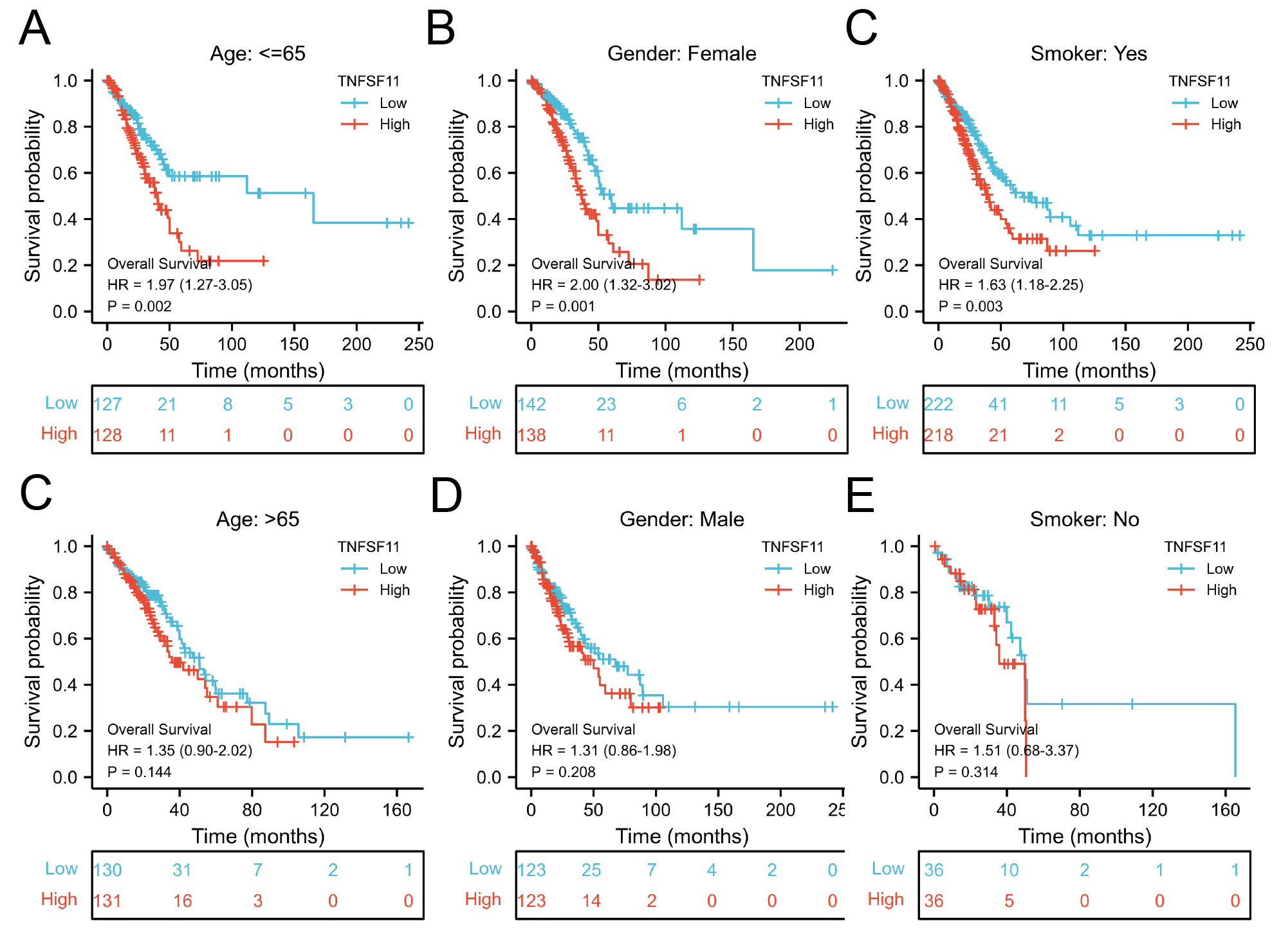

Supplement: Supplementary file 2 — Supplementary Material 2: Supplementary Fig. 2. Kaplan-Meier survival curves for the patients, (A and C) under the age of 65 years old and over 65 years old, (B and D) male and female, (C and E) Different smoking status. [file 12967_2024_5112_MOESM2_ESM.tif]

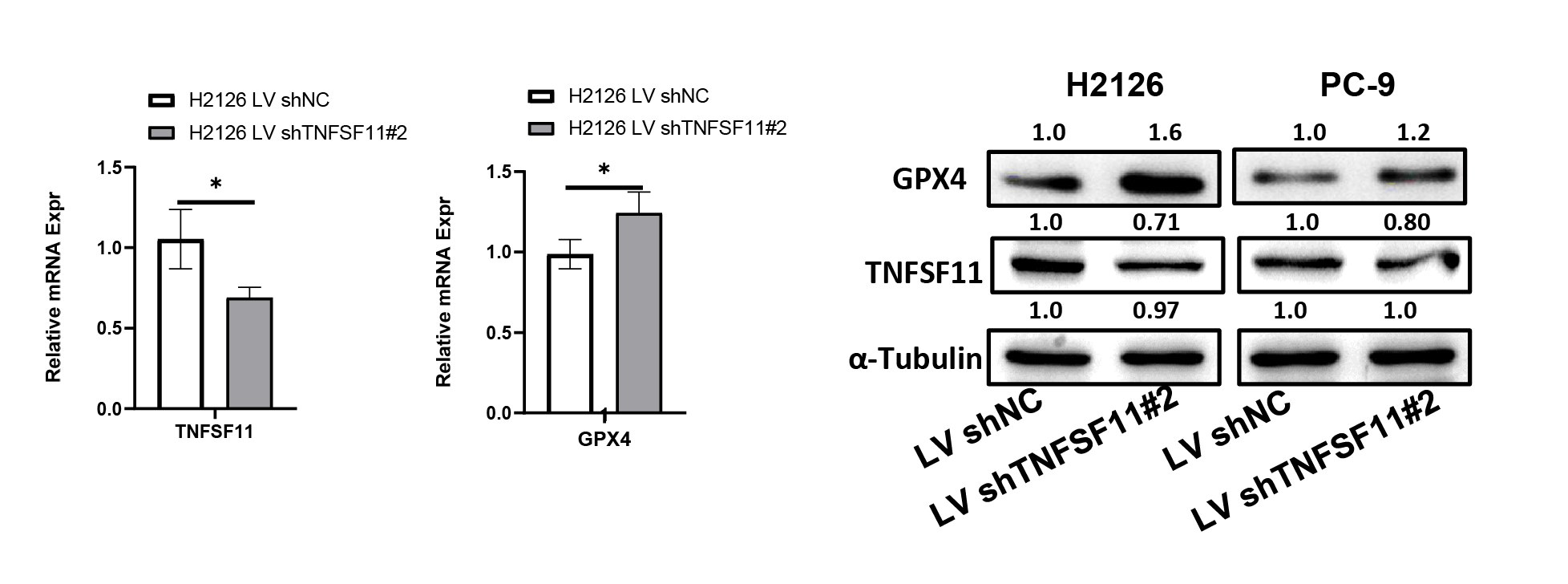

Supplement: Supplementary file 3 — Supplementary Material 3: Supplementary Fig. 3. TNFSF11 expression levels are correlated with GPX4. (A) Comparison of mRNA expression of TNFSF11 and GPX4 post lentiviral transduction with LV shTNFSF11 #2 in H2126 cells. Bars, means ± SD, n = 3. *P < 0.05. (B) Western blotting analysis of GPX4 and TNFSF11 expression in protein level following lentiviral transduction with LV shTNFSF11 #2 in H2126 and PC-9 cells. α-Tubulin served as the loading control. Each experiment was conducted in triplicate. [file 12967_2024_5112_MOESM3_ESM.tif]
